# Behavioral Responses of the Invasive Fly *Philornis downsi* to Stimuli from Bacteria and Yeast in the Laboratory and the Field in the Galapagos Islands

**DOI:** 10.3390/insects10120431

**Published:** 2019-11-28

**Authors:** Boaz Yuval, Paola Lahuatte, Polpass Arul Jose, Charlotte E. Causton, Edouard Jurkevitch, Nikos Kouloussis, Michael Ben-Yosef

**Affiliations:** 1 Faculty of Agriculture, Food and Environment, Hebrew University of Jerusalem, POB 12, Rehovot 761000, IsraelEdouard.jurkevitch@mail.huji.ac.il (E.J.); 2 Charles Darwin Research Station, Charles Darwin Foundation, Puerto Ayora, Galapagos 200350, Ecuador; paola.lahuatte@fcdarwin.org.ec (P.L.); charlotte.causton@fcdarwin.org.ec (C.E.C.); 3 Laboratory of Applied Zoology and Parasitology, School of Agriculture, Aristotle University of Thessaloniki, 54124 Thessaloniki, Greece; nikoul@agro.auth.gr; 4 Agricultural Research Organization, Gilat Center, M.P. Negev 85280, Israel; michaelb@volcani.agri.gov.il

**Keywords:** *Philornis*, invasive species, bacterial attraction, proboscis extension response

## Abstract

*Philornis downsi* Dodge and Aitken (Diptera: Muscidae) is an avian parasitic fly that has invaded the Galapagos archipelago and exerts an onerous burden on populations of endemic land birds. As part of an ongoing effort to develop tools for the integrated management of this fly, our objective was to determine its long- and short-range responses to bacterial and fungal cues associated with adult *P. downsi*. We hypothesized that the bacterial and fungal communities would elicit attraction at distance through volatiles, and appetitive responses upon contact. Accordingly, we amplified bacteria from guts of adult field-caught flies and from bird feces, and yeasts from fermenting papaya juice (a known attractant of *P. downsi*), on selective growth media, and assayed the response of flies to these microbes or their exudates. In the field, we baited traps with bacteria or yeast and monitored adult fly attraction. In the laboratory, we used the proboscis extension response (PER) to determine the sensitivity of males and females to tarsal contact with bacteria or yeast. Long range trapping efforts yielded two female flies over 112 trap-nights (attracted by bacteria from bird feces and from the gut of adult flies). In the laboratory, tarsal contact with stimuli from gut bacteria elicited significantly more responses than did yeast stimuli. We discuss the significance of these findings in context with other studies in the field and identify targets for future work.

## 1. Introduction

*Philornis downsi* Dodge and Aitken, 1968 (Diptera: Muscidae) is an avian nest parasitic fly that invaded the Galapagos archipelago in the latter half of the twentieth century [1,2,3,4]. Female flies oviposit in active bird nests. Post hatching, larvae undergo three instars, during which they typically feed on the nestlings present in the nest, first by acquiring blood from vessels in the host’s nares (during the first instar), and subsequently by nocturnal feeding bouts on blood and tissues of the host [3,5]. 

The broad host range of these flies, lack of competitors and natural enemies, and their high dispersal ability and adaptability to harsh environments have all contributed to their successful invasion [2]. This success is manifest in the impact on the local passerines. Since *P. downsi* was first observed in 1997 [6], nearly all the passerines on the islands have been recorded as hosts [2]. Furthermore, the intensity of parasitism (number of larvae per nest), has been rising, with attendant increase in mortality of the defenseless hosts. Particularly susceptible are species of the endemic (and iconic) group of birds known as “Darwin’s finches” [2,3]. Thus, for example, the medium tree-finch (*Camarhynchus pauper*) has lost >50% of its population on Floreana island [7]. Elsewhere, on the island of Santa Cruz, populations of the smaller tree-finch (*Camarhynchus parvulus*) are declining as levels of parasite infestation continue to rise [8]. On the island of Isabela, the last population of the critically endangered mangrove finch (*Camarhynchus heliobates*) is literally on the verge of extinction, due to the combined effects of predation, habitat loss, and *Philornis* parasitism [9].

Due to the protected status of the Galapagos Islands, a control approach based on indiscriminate application of insecticides is out of the question. Accordingly, an international consortium of researchers, coordinated by the Charles Darwin Foundation (CDF) and the Directorate of the Galapagos National Park in Puerto Ayora, Santa Cruz, is seeking to develop and implement strategies and tools for the management of *P. downsi* in the Galapagos Islands [10]. In the short term, these approaches consist of captive breeding of nestlings of the most endangered species [11], application of larvicide to nests [2,12], and trapping of adult flies [13]. The long-term vision is an integrated use of biological control with the sterile insect technique (SIT). Although several promising natural enemies have been identified in the Americas (e.g., [14]), implementation will take a while as the candidate enemies need to satisfy regulatory requirements prior to introduction into the fragile ecosystem of the Galapagos Islands. Concurrently, implementation of the SIT is hampered by vast lacunae in our understanding of the basic biology of *P. downsi*, as exemplified by the extreme difficulty of rearing this fly in captivity [15] and our lack of understanding about the particulars of its mating system and patterns of its behavior in the field [2]. 

Complementary to these approaches are methods to manipulate the microbiome of the target insect. The relationship between insects and microorganisms has received much attention in the past two decades, and hardly needs an introduction, as the contributions of symbionts to host nutrition, environmental adaptation, immunity, and ultimately, fitness, are quite well known [16]. In theory, once the specific microbial partners of an insect, and their effects on the host are identified, it is possible to manipulate them in a manner that benefits the goals of control operations (reviews in [17,18,19] and for examples see [20,21,22]). 

In flies (Diptera), gut and environmental bacteria have been found in many instances to provide cues that are important in foraging for food [23,24,25,26], oviposition sites [27,28,29,30,31], and mates [32]. Furthermore, volatiles from bacteria and fungi have been shown to attract flies [33] and to have potential for enhancing trap catches [34]. Indeed, Cha and colleagues [13], found that *P. downsi* were attracted to traps containing active bakers’ yeast (*Saccharomyces cerevisiae*) and in particular to acetic acid and the ethanol produced by the yeast. Currently, the standard method of capturing *P. downsi* in the field is with McPhail traps baited with fermenting papaya juice [35]. This attractant is superior to commercially available fly attractants, yet it is cumbersome and many non-target flies, wasps, and moths are trapped, together with less than one *P. downsi*/trap/day. This method provides an index for the population, but does not reflect the true population size, as there is no direct correlation between number of larvae in nests and flies caught in traps during long-term monitoring [35]. Furthermore, intensive trapping fails to reduce abundance, as they apparently fail to compete with environmental cues such as flowering and fruiting plants. Clearly a selective, efficient trap would be a welcome addition to the tools employed for the study and control of this fly.

We believe that there are several possible uses for microbes in *P. downsi* management such as improving diets for mass rearing for SIT and for developing effective attractants for use in traps. Recently, we characterized the bacterial microbiome of *P. downsi* [36]. We found that larval and adult microbiomes are dominated by the phyla Proteobacteria and Firmicutes, with communities that significantly differ between life stages, reflecting the different dietary needs of the larvae and adults. In light of the widespread importance of the microbiome in shaping behavior of many insects (references above, also see [37,38]), we hypothesized that the bacterial and fungal communities associated with adult *P. downsi* will elicit attraction at distance through volatiles, and appetitive responses upon contact. Accordingly, we amplified bacteria from guts of adult field-caught individuals, and from bird feces (which may be a dietary resource for the flies, and serve in host location), and yeast from fermenting papaya juice, on selective growth media, and assayed the response of flies to these microbes or their exudates. In the field, we baited traps with bacteria or yeast and monitored adult fly attraction. In the laboratory, we used the proboscis extension response (PER) (e.g., [24,39]) to determine the sensitivity of males and females to tarsal contact with bacteria or yeast.

## 2. Materials and Methods 

### 2.1. Traps and Baits

**Figure 1:**
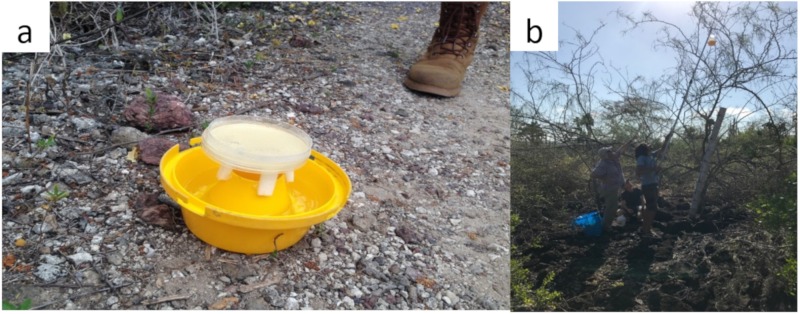
(**a**) Modified McPhail trap with bacterial bait (trap cover not shown) and (**b**) traps positioned at El Barranco area (Santa Cruz Island, Galapagos). The traps were observed for flies/insects after a trapping night. Concurrently, consortia of bacteria and yeasts from isolation plates were collected for identification using next generation amplicon sequencing.

We devised a modified McPhail trap to hold a 10 cm Petri dish inoculated with bacteria or yeast (Figure 1). We deployed these traps along transects at two locations on the island of Santa Cruz: El Barranco (−0.738117; −90.301656), a lowland, arid site and Los Gemelos (−0.626379; −90.380697), a highland, humid site [35,40]. These are areas where *P. downsi* is commonly collected. Traps were deployed overnight on 8, 9, 10, 14, and 15 February and 7, 8, and 9 March 2018, and on 11, 13, and 15 February 2019, for a total of 112 trap-nights (Table 1). These periods fell within the nesting season of the local finch species [41]. Traps were placed ~3–5 m high on branches of trees and spaced at least 10 meters apart. Traps were positioned 2 h before sunset and recovered 2–3 h after sunrise the following day. This allowed for catching flies at dawn and dusk, times when flies are most active [2]. 

To assay attraction to bacterial volatiles, we dissected the gut of 4–6 freshly caught female and male *P. downsi*, homogenized the gut contents in phosphate buffered saline (PBS), and inoculated them on plates with a selective growth medium; either Luria–Bertani (LB), tomato (TA), or brain heart (BH) agar (BD Difco). Twenty-four hours following the inoculation, the growth plates, with the bacterial colonies, were positioned (uncovered) as bait in the traps. In addition to bacteria from flies, we also cultured bacteria from fresh feces of a medium ground-finch (*Geospiza fortis*) on LB plates and baited traps in a similar manner. To assay attraction to yeast volatiles, a sample from fermenting papaya juice (routinely used for baiting traps) was inoculated on yeast-extract potato dextrose (YPD, BD Difco) agar plates supplemented with chloramphenicol (50 µg/mL) for suppression of bacteria. Bacterial and yeast communities developing on media plates were subsequently identified, respectively, by sequencing the 16S rRNA and ITS genes (see following section).

### 2.2. Determination of Community Composition of Bacteria and Yeasts

Genomic DNA was isolated from the bacterial and fungal consortia harvested from the different media plates we used (LB and TA for bacteria and YPD for yeasts). DNeasy Blood and Tissue Kit (Qiagen, Hilden, Germany) was used for the isolation of DNA with sample-specific pre-treatment according to the manufacturer’s instructions. From the isolated bacterial DNA, the V4 region of 16S rRNA gene was PCR amplified with eubacterial primers 515F and 806R [42,43,44]. For the fungal ITS region, the standard ITS primer pair (ITS1F–ITS2) was used with genomic DNA extracted from yeast consortium as template. Bacterial 16S and fungal ITS amplicon libraries were prepared and sequenced on the Illumina MiniSeq platform at Sequencing Core (SQC), University of Illinois Chicago, as described elsewhere [45]. Resulting raw Illumina read pairs were quality trimmed using BBDuk with standard parameters (https://jgi.doe.gov/data-and-tools/bbtools/bb-tools-user-guide/bbduk-guide/). Resulting reads were merged, and quality filtered using Mothur v.1.42.1 [46]. After removing chimeric reads, OTU clustering was done with a 97% sequence identity threshold and sequences were assigned under different taxonomic groups according to the latest SILVA taxonomy release v.132 [47]. For ITS reads processing, Mothur SOP was adopted with modifications in make contigs (delseq = 0) and screen seqs commands (maxhomopol = 12) to suit fungal sequence data. OTU clustering was done with a 95% sequence identity threshold and taxonomic classification was done with reference dataset distributed by UNITE [48]. The sequence data is publicly available on the MG-RAST platform: https://www.mg-rast.org/mgmain.html?mgpage=project&project=mgp91882.

### 2.3. Proboscis Extension Response Assay

The PER experiment was done on two populations of flies. The first were adults that emerged from pupae that were collected from nests after fledglings had left the nest or died. The nests were brought back to the laboratory and pupae removed from the nest material. Following emergence, flies were held in sex specific cages (dimensions: 45 × 45 × 45 cm) and provided with ripe berries of Muyuyo (*Cordia lutea,* Boraginaceae) and water *ad lib*. On the day of the experiment they were 6–9 days old.

The second group were adult flies caught in McPhail traps baited with fermented papaya juice that were placed around the Charles Darwin Research Station (CDRS) in Puerto Ayora (−0.741425; −90.302766), and brought back to the laboratory between 26 January 2019 and 2 February 2019. These flies were at least 14–21 days old on the day of the experiment (15 February 2019). Following capture, they were held in cages as described above, and fed on a mixture of pulped papaya (34.2%), protein powder (2.6%), egg powder (3%), milk powder (2.6%), sugar (7.9%), and water (50%). 

**Figure 2:**
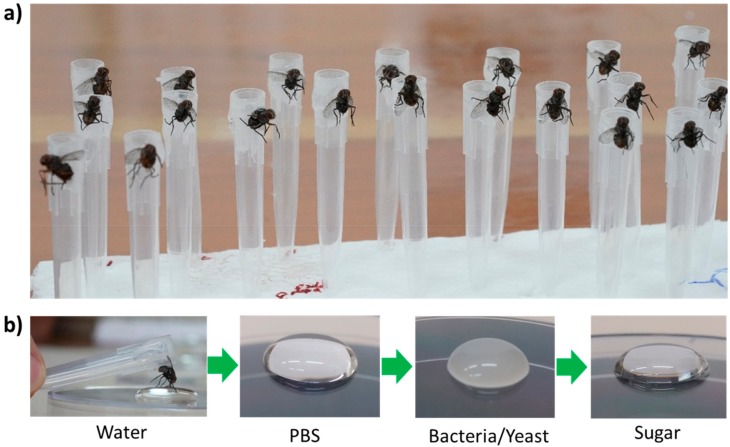
Overview of proboscis extension response (PER) experiment. (**a**) *Philornis downsi* adults were glued with liquid silicon to the base of a disposable pipette tip. (**b**) Subsequently the flies were individually assessed for their proboscis extension response to a sequence of stimuli.

Four hours prior to the experiment, flies were attached with liquid silicon to the base of a disposable pipette tip and allowed to rest in a humidified environment (Figure 2a). 

The assay itself consisted of presenting the tarsi of the flies with a series of stimuli and recording the response of the proboscis. The stimuli were presented as droplets in the following order: Water (allowing flies to drink their fill), phosphate buffered saline (PBS, negative control), bacteria or yeast suspensions in PBS (test stimulus), and 20% sucrose solution (positive control) (Figure 2b). Apart from water, flies were allowed 5 s of tarsal contact with each stimulus. Test stimuli were prepared by suspending bacterial or yeast colonies developing on agar media (LB and YPD, respectively) in sterile PBS. Microorganisms originated from the gut homogenate of flies that were freshly caught in the field (bacteria) or fermenting papaya juice (yeast). Care was taken to present the flies with dense microbial suspensions overabundant in cells in order to saturate tarsal sensilla with possible stimuli emanating from microbes. Full extension of the proboscis in response to tarsal contact with the test stimulus (bacteria or yeast) was scored as a positive result. Flies responding to the negative control (PBS, five females from pupae) and those *not* responding to the positive control (sucrose solution, three females and three males derived from pupae, and two trapped females) were excluded from the analysis.

### 2.4. Statistical Analysis

The results of the PER experiment were analyzed using a generalized linear model (GLM) with binomial distribution and log link function (JMP Pro version 14, SAS Institute Inc., Cary, NC., USA). Microbiome data were relativized according to the sum of read abundance in each sample. Statistical analysis was performed with the multiresponse permutation procedure (MRPP) using Bray–Curtis distances.

## 3. Results

### 3.1. Community Composition of Bacterial and Yeast Baits Used in Assays

**Figure 3:**
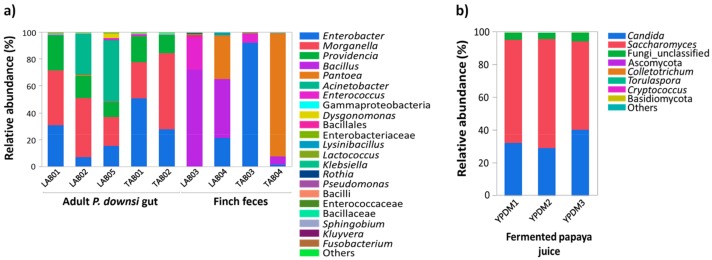
Composition of bacterial (**a**) and fungal (**b**) consortia acquired from adult *P. downsi* gut or finch feces. Consortia were used in traps and as stimuli (LAB05; YPDM1) in the PER experiment. Color codes of bacterial and fungal genera with relative abundance of >0.1(%) are listed in the right-side panels. LAB01-05: Lysogeny agar, TAB01-04: Tomato agar, and YPDM1-3: Yeast-extract potato dextrose media.

The community composition of culture-dependent bacteria and yeasts, which were used as baits in traps and as stimuli in the PER experiment is shown in Figure 3. 

The gut microbial communities developing on LB and TA plates did not differ significantly (MRPP test, A = −0.009, *p* = 0.23 after Bonferroni correction). These communities were dominated by *Morganella* (Figure 3a; mean ± SD: 35.5% + 12.2%), *Providencia* (17.8% ± 7.4%), *Enterobacter* (17.8% ± 12%), and *Acinetobacter* (25.3% ± 22.4%). Notably, this community structure differed from the bacteria acquired on agar plates inoculated with finch feces (MRPP test, A = 0.228, *p* = 0.001 after Bonferroni correction). In fecal samples *Bacillus* (57.9% ± 20.11%), *Pantoea* (16.09% ± 22.7%), and *Enterococcus* (11.7% ± 16.6%) were dominant on LB, while either *Enterobacter* (46.9% ± 64.2%) or *Pantoea* (45.7% ± 64.6%) dominated the TA plates.

The yeast communities used in the trapping and PER experiment were dominated by species of *Saccharomyces* (Figure 3b, 61.5% ± 6.6%) and *Candida* (33.9% ± 5.7%).

### 3.2. Trapping

Over a total of 112 trap-nights, we caught only two *P. downsi* females (Table 1). One was attracted by gut bacteria cultured on LB, and the other by bacteria originating from finch feces and cultured on LB media. Interestingly, a variety of other flies were caught, mainly sarcophagids, calliphorids, and tephritids suggesting that bacterial volatiles were generally attractive to dipterans. In some cases, there was a higher number of flies caught in the controls suggesting that they were attracted to the growth media. Also of interest was the observation that few other insects, namely wasps and moths, were attracted to traps in the Los Gemelos site (Table 1). 

### 3.3. PER Experiment

**Figure 4:**
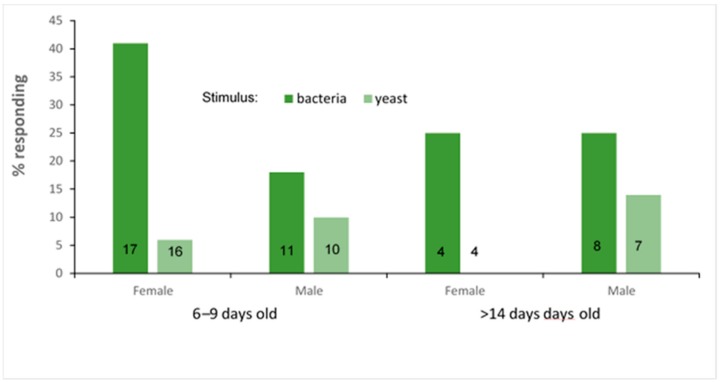
Proboscis extension response of *P. downsi* to bacterial or yeast stimuli. Flies 6–9 days old were derived from field-collected pupae recovered from naturally-infested nests. Flies >14 days old were trapped as adults in the field and maintained in the laboratory prior to testing. The difference in the response is highly significant (GLM effect test x^2^ = 5.96, *p* = 0.014). Numbers within columns denote sample size (note that none of the four older females responded to yeast).

The PER experimental results were more conclusive (Figure 4). Bacteria cultured from the guts of a freshly caught female elicited significantly more responses than yeasts cultured from fermented papaya. Overall, 38% of females (n = 21) and 21% of males (n = 19) responded to bacteria, while only 5% of the females (n = 20) and 11% of the males (n = 17) responded to the yeast (Figure 4, note that none of the older females responded to yeast). The whole model analysis, which included the stimulus tested, the source of the flies (trapped as adults or emerged from pupae collected from nests) and their sex, was marginally significant (GLM maximum likelihood x^2^ = 6.31, *p* = 0.09, n = 77). This model also tested the effects individually and revealed that there was a significant difference in the reaction to the stimulus (bacteria or yeast) (x^2^ = 5.96, *p* = 0.014), while the source of the flies (x^2^ = 0.035, *p* = 0.85), and their sex (x^2^ = 0.06, *p* = 0.79), were not significant in eliciting a different response to bacteria or yeast.

On excluding the source of the flies (which showed no differences) from the analysis, the picture becomes even stronger (general linear model x^2^ = 6.6, *p* = 0.035, n= 77). While there were still no differences between the responses of males or females (x^2^ = 6.3, *p* = 0.51), the response to the stimuli differed significantly (x^2^ = 34.5, *p* < 0.0001), with more flies responding to stimuli from bacteria compared to yeasts. 

## 4. Discussion

The development of a selective and efficient trap for *P. downsi* has been identified as a research priority and would greatly enhance future integrated management programs [10]. In light of published studies of attraction of other flies to microbial volatiles (see introduction), we had high hopes for our novel modified traps. Sadly, we cannot but conclude that this approach has failed. Previous studies aimed at assaying various attractants for this fly resulted in much higher catches per trap. Lincango and Causton (unpublished report, CDF 2009) review attractants that were tested between 2007 and 2009; among them, milk powder, Biolure, tricocene, muyuyo berries (*Cordia lutea*), amines, indole, putrecine, methyl-amine, and fermented papaya juice. The latter proved to be the strongest attractant, and is currently used in all monitoring efforts [35]. Recently Cha et al., [13] identified a number of attractive volatiles from fermenting baker’s yeast, pinpointing acetic acid and ethanol as the most potent. The most attractive bait they assayed was a liquid combination of yeast and sugar. Extrapolating from the results of both these studies indicates that the best attractants (fermented papaya juice and active yeast), yield captures approaching one fly/trap/day. Our results come nowhere near, and even if we consider that in 2018, our traps were deployed very early in the nesting season, this cannot be said for 2019, when nesting was in full swing and papaya-baited traps were capturing many flies. We left our traps out overnight, but they were available to the flies for at least 2 h in the evening, and 2–3 h in the morning, so (like the many flies from other species) they had ample opportunity to access the baits. Indeed, on the few occasions when traps were not collected immediately, results were the same. 

Interestingly, one of the females we caught was in a trap baited with bacteria from the feces of a medium ground-finch (*Geospiza fortis*). The feces we used were from a fresh deposit made by an individual frequenting the cafeteria at CDRS. In our culture-dependent samples, community structure in feces was significantly less diverse than the communities found in flies. A recent study by Knutie et al. [49] examined the effect of proximity to humans on the microbial community in finch feces. Using a culture-independent approach, they found that proximity to humans lowered the diversity of the microbiome in this species. 

The other female we trapped was attracted to a community of gut bacteria from adult flies growing on LB medium. The analysis of this community (Figure 3) corresponded to a previously published culture-independent metagenomic study [36]. The community contained taxa such as Enterobacteriacae that in other insect species are known to be important in attraction [28,34,37]. Our results do not immediately suggest that this microbial community attracts foraging flies. However, the mechanism whereby female flies localize host nests or food sources is as yet unknown, and a microbial dimension may have an important (as yet unrevealed) part to play. That said, our result of two flies in 112 trap-nights is sobering (these may very well be the two most expensive flies in the history of entomology) and a change in our methodology is mandated. Nevertheless, we feel it is important to share our results, and welcome any suggestions for improving our approach. 

The PER has been used widely to characterize the sensitivity of chemical receptors of flies and bees [50,51,52] and as a conditioned response in learning trials [39,53,54,55]. In our experiment, we tested the response of adult flies to a suspension containing either bacteria derived from the gut of an adult female fly, or yeast growing in fermented papaya juice. The response to sucrose at the end of the test unequivocally shows that all flies were motivated to ingest food, and the exclusion of flies responding to PBS assures us that the responses we considered were indeed specific to the test stimuli (bacteria or yeast). 

We used flies from two populations in this experiment, young individuals reared from pupae collected from nests, and older flies that were trapped in the field at an unknown age and maintained for 14–21 days in the laboratory. Although both responded in a similar manner to the stimuli, such that it was indistinguishable statistically, the trend observed was that the older flies were less responsive overall (Figure 4). Apart from their age, these groups differed in the adult diet they received, and possibly in sexual experience. The young group were all virgins, while there is a high probability that the older group had mated prior to capture. The microbiome of flies is known to affect their responses to nutritional cues [23,24,26,38]. Different diets support different gut microbial communities, which in turn may affect behavior in a different manner. It may very well be that the different diets ingested in the laboratory (and previously in the field, by the older flies) affected the magnitude of the responses observed, if not their direction. An ongoing study using culture-independent approaches focuses on the effect of diet and colonization on bacterial consortia in *Philornis*, and the effect of these regimes on appetitive behavior.

We found that both females and males (but especially the females) from the two populations we assayed showed a significantly higher response to the bacterial cues. Thus, although flies responded to volatiles from active yeasts and fermented papaya juice [13], suspended yeasts rarely elicited proboscis response following contact with the flies’ tarsi. The elevated response to bacterial cues is intriguing. All responding flies were eager to eat, and the obvious conclusion is that the bacterial cue is associated with a food substrate. Although we do not know exactly what the main sources of nutrition are it is reasonable to assume that *P. downsi* are polyphagous and ingest decaying organic matter, fruit juices, and possibly pollen and nectar [2,3,5], as well as (in the laboratory), bird feces (unpublished observation). While many of these substrates are associated with bacteria, they are frequently also associated with yeasts, which elicited a significantly lower response. Microbial interactions within the fermentation broth may directly affect metabolite production or yield volatiles that are not present on culture plates [56]. Clearly, more work needs to be done to identify if the flies are responding to a specific bacterial species or metabolic product, and how the experiential and symbiotic status of the fly influences its responses. We suggest that the PER paradigm will be extremely useful in the future to answer these questions.

## 5. Conclusions

Our study examined responses of *P. downsi* to microbial volatiles and tactile cues. We found no evidence for long-range attraction to microbial volatiles, yet recognize that this may be due to a flaw in our trap design. Conversely, tethered flies exhibited a feeding response to bacterial cues with alacrity, significantly more so than to fungal cues. Future work will continue to investigate the importance of both bacteria and fungi in the nutritional ecology of this invasive fly.

## Figures and Tables

**Table 1:** Long Range Attraction to Microbial Volatiles: Trapping Results.

Location	Number of Trap-Nights	Bait/Medium	No. of *Philornis*	Mean Number (SD) of Other Flies/Trap	Other Insects Trapped
El Barranco	20	bacteria/LB	1 (female)	4.6 (5.2)	1 ant, 3 wasps
El Barranco	8	finch feces/LB	1 (female)	5.2 (2.7)	8 ants
El Barranco	3	control/LB	0	5.6 (5.1)	1 wasp
El Barranco	12	bacteria/TA	0	14.6 (16.2)	2 ants, 3 wasps
El Barranco	3	control/TA	0	15.6 (16.8)	1 wasp
El Barranco	8	bacteria/BH	0	3.3 (1.9)	3 ants, 21 wasps, 3 moths
El Barranco	2	control/BH	0	1 (1.4)	2 wasps
El Barranco	8	yeasts/YPD	0	0.3 (0.7)	1 ant, 1 wasp
El Barranco	2	control/YPD	0	1.5 (2.1)	1 ant 1 wasp
Los Gemelos	16	bacteria/LB	0	1.8 (1.6)	0
Los Gemelos	4	bacteria/LB	0	2 (2.1)	0
Los Gemelos	16	bacteria/TA	0	2.8 (2.5)	1 moth
Los Gemelos	8	fermented papaya/TA	0	1.8 (1.5)	1 cockroach
Los Gemelos	2	control/TA	0	7.5 (4.9)	0

Table 1: Summary of insect catches in modified McPhail traps. Traps were baited with live colonies of bacteria (from adult fly guts or finch feces), yeast, or fermented papaya juice. Media for microbial growth were: LB- Luria Bertoni; TA- Tomato Agar; BH- Brain Heart Infusion; YPD- yeast-extract potato dextrose.
